# An Unusual First Presentation of Colon Cancer: Pulmonary Lymphangitic Carcinomatosis

**DOI:** 10.7759/cureus.103238

**Published:** 2026-02-08

**Authors:** Jenny Joseph, Brandon Bharat, Hira Bakhtiar

**Affiliations:** 1 Internal Medicine, Norwalk Hospital, Norwalk, USA; 2 Internal Medicine, Ross University School of Medicine, Bridgeport, BRB; 3 Pulmonary and Critical Care Medicine, Norwalk Hospital, Norwalk, USA

**Keywords:** colon cancer, interstitial lung disease, lung metastases, occult malignancy, pulmonary lymphangitic carcinomatosis

## Abstract

Pulmonary lymphangitic carcinomatosis (PLC) is a rare manifestation of metastatic disease that presents with nonspecific respiratory symptoms. We describe a young male who presented with progressive dyspnea and cough and was initially managed for pneumonia. Chest imaging demonstrated diffuse interstitial abnormalities with mediastinal lymphadenopathy and pleural effusions, prompting evaluation for interstitial lung disease (ILD). Extensive infectious and rheumatologic testing was unrevealing. Cytologic analysis of pleural fluid revealed adenocarcinoma, and subsequent imaging identified a primary sigmoid colon malignancy. Despite the initiation of chemotherapy, the patient rapidly deteriorated. This case underscores the diagnostic challenges of PLC and emphasizes the importance of maintaining a broad differential diagnosis in patients with persistent respiratory symptoms and imaging findings similar to ILD. It also highlights the importance of considering occult colorectal malignancy in cases of PLC.

## Introduction

Pulmonary lymphangitic carcinomatosis (PLC) is a rare manifestation of lung metastases, accounting for 6% to 8% of intrathoracic metastases [1]. Breast, gastric, and lung cancers are the most associated primary tumors [2]. It is characterized by the presence of a tumor in pulmonary lymphatic vessels predominantly manifesting as dyspnea [2,3] and non-productive cough [4]. Pulmonary metastases are rare with colorectal carcinomas. Colon cancers most commonly metastasize to the liver through the portal vein, which drains the colon, whereas rectal cancers can metastasize to the lungs due to the dual venous drainage into the inferior vena cava [4]. This case highlights a rare presentation of occult primary colon cancer and the importance of considering PLC in the differential diagnoses despite the inconspicuous presentation.

## Case presentation

**Figure 1:**
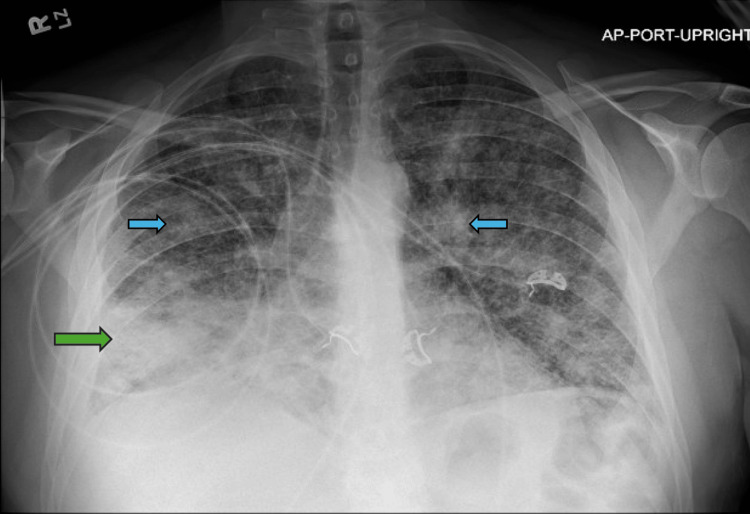
The CXR showing bilateral reticulonodular opacities (blue arrows) and right lung consolidation (green arrow) CXR: Chest X-ray

A 38-year-old male with a history of e-cigarette usage and childhood asthma presented with intractable cough and dyspnea that began almost three weeks prior to presentation. Family history was negative for cancer. Examination revealed bilateral scattered crackles. The initial chest X-ray (CXR) showed bilateral reticulonodular opacities with right lung consolidation (Figure 1). He was treated for pneumonia with no significant improvement despite antibiotics. He subsequently underwent bronchoscopy with bronchoalveolar lavage (BAL). The BAL analysis (Table 1) revealed polymorphonuclear neutrophils (PMNs) suggestive of inflammation or infection, but cultures were persistently negative. The BAL cytology revealed atypical cells with degenerative changes in a background of benign bronchial cells and mixed inflammatory cells. Infectious and rheumatologic workups, including interferon-gamma release assay (IGRA), Mycobacterium tuberculosis (MTB) PCR, acid-fast bacilli (AFB) cultures, and serum angiotensin-converting enzyme (ACE) levels, resulted in negative findings and were notable only for mildly positive antinuclear antibodies (ANA) (Table 2).

**Table 1:** The BAL studies BAL: Bronchoalveolar lavage

Specimen	Bronchoalveolar lavage
Appearance	Cloudy
Color	Colorless
WBC	283/cumm
RBC	<3000/cumm
Polymorphonuclear cells	93%
Lymphocytes	6%
Monocytes	1%
Culture	No growth
Cytology	Atypical cells with degenerative changes, benign bronchial cells, and mixed inflammatory cells

**Table 2:** Laboratory workup ANA: Antinuclear antibodies, ACE: Angiotensin-converting enzyme, IGRA: Interferon-gamma release assay, AFB: Acid-fast bacilli

Test	Result	Reference range	Interpretation
ANA titer	1:320	Normal low <1:80	Positive
ACE level	17 U/L	11-80 U/L	Normal
IGRA	Negative	Positive/negative/indeterminate	Negative
Blood and lower respiratory cultures, including fungal and AFB	Negative	Positive/negative	Negative

**Figure 2:**
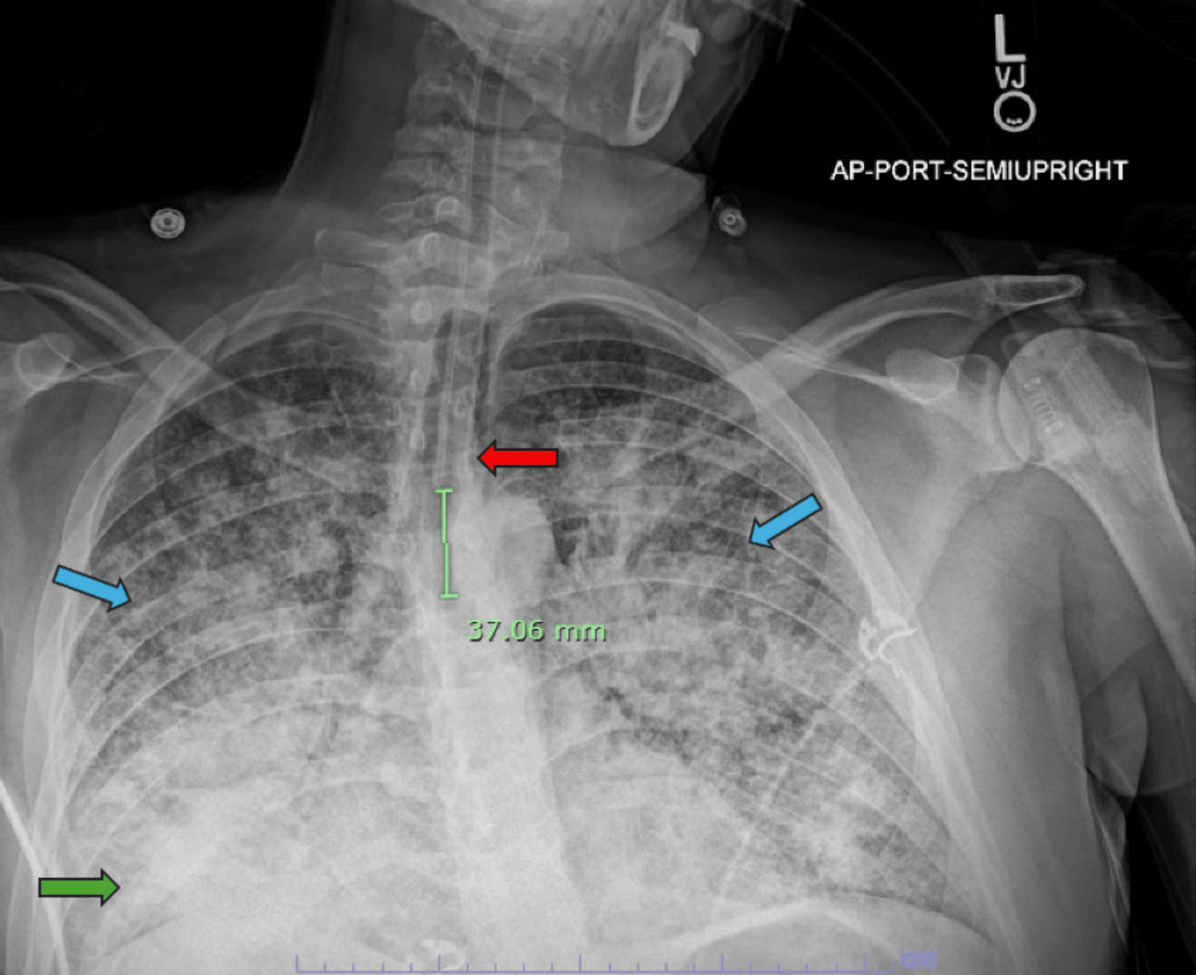
Repeat CXR showing worsening right lower lobe consolidation (green arrow) and bilateral reticulonodular opacities (blue arrows) with endotracheal tube (red arrow) terminating 3.7 cm above the carina CXR: Chest X-ray

**Figure 3:**
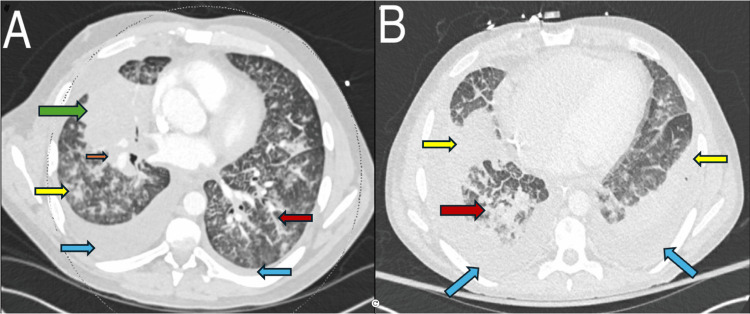
Contrast-enhanced CT of the chest (A) and and abdomen/pelvis (B) A: Contrast-enhanced CT chest showing right lung consolidation (green arrow) with nodular opacities (yellow arrow) and interlobular septal thickening (red arrow), mediastinal lymphadenopathy (orange arrow), and pleural thickening; B: Repeat contrast-enhanced CT scan chest/abdomen/pelvis showing worsening multifocal airspace disease (red arrow) (thickened interlobular septa with ground-glass opacities), bilateral consolidations (yellow arrows), and pleural thickening with new bilateral pleural effusions (blue arrows)

A repeat CXR showed bilateral reticulonodular opacities and worsening right lung consolidation (Figure 2). Further, contrast-enhanced CT chest revealed interlobular septal thickening, mediastinal lymphadenopathy, and pleural thickening (Figure 3, panel A). Later, the patient required mechanical ventilation for acute hypoxic respiratory failure. After successful extubation, he was discharged on steroids and supplemental oxygen for possible acute interstitial pneumonia (AIP) of unclear etiology. 

Approximately one week following discharge, the patient was seen in the pulmonology clinic for follow-up and was noted to be in significant respiratory distress, necessitating transfer to a tertiary care facility. Repeat contrast-enhanced CT chest/abdomen/pelvis showed diffuse lymphadenopathy, multiple osteolytic lesions, worsening multifocal airspace disease (thickened interlobular septa with ground-glass opacities), pleural thickening with new bilateral pleural effusions (Figure 3, panel B), ascites, and sigmoid colon thickening. He underwent thoracentesis, and pleural fluid cytology was consistent with metastatic adenocarcinoma, suggestive of metastases through lymphatics into the pleural space. The clinical picture was suggestive of PLC. Sigmoidoscopy revealed a mass in the colon, which was biopsy-proven to be adenocarcinoma with lymphovascular invasion, and genetic analysis revealed mutant BRAF V6005. Although he improved on chemotherapy with folinic acid, fluorouracil, and oxaliplatin (FOLFOX), cetuximab, and encorafenib, he developed acute respiratory failure and succumbed to his illness almost six months from initial presentation. 

## Discussion

Pulmonary lymphangitic carcinomatosis shares several clinical features with ILD, such as AIP, as both commonly present with symptoms of dyspnea and cough, which made ILD a leading differential in our case. The CXR findings suggestive of PLC include diffuse reticulonodular infiltrates, Kerley B lines, hilar lymphadenopathy, and occasionally pleural effusions [5]. Characteristic CT chest findings consist of hilar and mediastinal lymphadenopathy and thickening of interlobular septa, along with ground-glass opacities reflecting interstitial edema, as observed in our patient [4]. While imaging characteristics of PLC and ILD may overlap, lack of clinical improvement with bronchodilators, antibiotics, and corticosteroids should prompt consideration of PLC. 

Differential diagnoses can include miliary tuberculosis, especially if patients present with symptoms like hemoptysis and weight loss. The IGRA test, AFB cultures, and MTB PCR help rule out tuberculosis. Another possible etiology is sarcoidosis, which can be screened for by checking ACE levels [6]. As these tests were negative in our patient, tuberculosis and sarcoidosis were ruled out. Lung biopsy is the diagnostic test of choice in PLC, but it might not be feasible in high-risk patients [7]. In such cases, BAL analysis may be helpful, but it is not sensitive. In our patient, BAL was inconclusive, and a diagnosis was made based on pleural fluid cytology. The mainstay of treatment in PLC is treatment directed toward the underlying primary malignancy to achieve cytoreduction. 

Pulmonary lymphangitic carcinomatosis has a poor prognosis with approximately 50% mortality in the first two to three months [1,8]. It can sometimes be the initial manifestation of an occult neoplasm, most commonly in individuals aged 40 to 49 years [1]. This case highlights the importance of recognizing the colon as a possible occult primary in cases of PLC. Otsubo et al. reported a case highlighting that colon adenocarcinoma with a micropapillary component is more prone to lymphovascular spread [9], resulting in PLC. Standard chemotherapy regimens have less efficacy in metastatic colon cancer with mutant BRAF V600E, in which improved survival was noted with FOLFOX-cetuximab and encorafenib [10]. Despite receiving appropriate chemotherapy, our patient’s condition did not improve. 

## Conclusions

Pulmonary lymphangitic carcinomatosis is an aggressive and often underrecognized manifestation of metastatic disease that may be the first clue to an occult malignancy. This case highlights the importance of maintaining a high index of suspicion in patients with persistent respiratory symptoms who fail to improve with standard therapies, including antibiotics and steroids, especially because imaging findings overlap with ILD. Early consideration of pulmonary lymphangitic spread and prompt investigation for an underlying primary cancer, including colorectal malignancy, are essential, as delays in diagnosis can significantly impact outcomes given the rapidly progressive nature and poor prognosis of this condition.
